# Scanning electron microscope analysis of elements in amalgam restorations to determine when a deceased person may have lived. A method based on data from Scandinavia

**DOI:** 10.1007/s00414-025-03561-8

**Published:** 2025-07-04

**Authors:** Sandra E. Floberg, Dalia Shwan, Thuyen Michelle Tran, Sigrid I. Kvaal, Jon E. Dahl, Simen E. Kopperud

**Affiliations:** 1 Faculty of Dentistry, University of Oslo, Oslo, Norway; 2 Nordic Institute of Dental Materials (NIOM), Oslo, Norway

**Keywords:** Forensic dentistry, Human identification, Dental amalgam, Electron scanning microscopy

## Abstract

When unidentified human remains are found, the aim must be to establish the identity of the individual. Being able to assess the time period when the deceased person has lived, might narrow down the search among missing persons. Dental amalgam alloys contain different elemental metals, and various alloy types have over time been introduced and abandoned at certain times, especially in Scandinavian countries. This study aimed to explore if a scanning electron microscope (SEM–EDS) can be used to determine the chemical composition of different dental amalgam restorations, with the focus to distinguish between copper amalgam and conventional silver amalgam. Six previously commonly used amalgams were selected, and five test samples were prepared from each of them. Each test sample was analysed with SEM–EDS at five separate sites, giving 25 readings for each amalgam brand. The SEM–EDS analyses identified noticeable differences in the composition of conventional silver amalgams compared to copper amalgams. The latter contained very low- or non-detectable amounts of silver and tin. The results showed that SEM–EDS could be a valuable accessory for identifying an unknown human remain.

## Introduction

When unidentified human remains are found, the aim is to establish the identity of the deceased individual. In some cases, it might be helpful to assess the time period during which the deceased person may have lived. Dental identification is in most cases established based on dental restorations seen in the victim’s dentition compared with that registered in a missing person’s dental records [1]. If the remains include teeth that contain amalgam restorations, it is obvious that the person has lived when or where that dental restorative material was in use. The development and advancement of modern dental amalgam was initiated in France already early in the 19th century [2, 3]. Thus, the discovery of an amalgam restoration among the remains does not necessarily imply that the deceased person has lived recently.

The literature describes three main groups of amalgam; low-copper amalgams (≤ 6 wt% copper), high-copper amalgams (≥ 6 wt% copper), and the so-called “copper amalgams” [4]. The first two types described are also typically referred to as "silver amalgams". The latter refers to a copper-based alloy consisting of 60–70% mercury and 30–40% copper. A characteristic trait of copper amalgam was that it did not contain any silver, but that it could contain other metals [5].

The first copper amalgam was introduced in 1859 [6]. However, the use of the material reached a peak in Norway and Sweden during the post-war years especially in caries active children, due to its documented superior cariostatic effect compared with conventional silver amalgams [7]. Commonly used copper amalgam products were “Cupromuc” and “Neo Silbrin” [8]. The copper amalgams had to be heated in a spoon over an open flame until droplets of mercury were visible on the surface, Then the material was triturated in a glass mortar and allowed to cool prior to filling the cavity [9]. In 1978 the FDI World Dental Federation advised specifically against heating amalgams due to health concerns from exhaust gases [10]. Some years later, in the 1980 s, the use of copper amalgam was abandoned in Scandinavia due to substantial corrosion rate, leading to the release of metallic ions, including copper, metallic mercury, and, in certain instances, the toxic heavy metal cadmium [5]. In addition, the corrosion caused considerable substance loss clinically and severe discoloration of surrounding tooth structures. The Norwegian Directorate of Health published in 1981 a governmental advice to show the greatest restraint towards the use of copper amalgam [11]; an advice that was regarded as a prohibition by most dentists. In Sweden, use of copper amalgam was prohibited in 1987 [11]. As a result, copper amalgam has a defined time period when patients received such treatment in Norway and Sweden, with a defined peak of use in the post-war years until the 1980s.

A scanning electron microscope (SEM) can be used to obtain information about the surface of a material and is one of the most used techniques in the characterization of biomaterials [12]. The elemental composition of the surface can be determined using SEM–EDS elemental analysis, or «energy-dispersive X-ray spectroscopy», which refers to the process where the energy of the emitted X-rays is measured and used to identify the elements in the sample [13]. SEM–EDS elemental analyses of dental restorations has previously shown to be a valuable tool for forensic odontologists in other parts of the world. In the Crash of Colgan Air Flight 3407 in 2009, SEM–EDS analyses were employed to assess compositions of both resin composites and endodontic sealers, which were essential for the identification of two of the victims [14]. SEM–EDS has also been useful for forensic purposes in analysing and identifying specific dental restorations before and after exposure to high temperatures [15, 16].

### Outline of problem

This study aims to explore if scanning electron microscope (SEM) with SEM–EDS elemental analysis can be used to determine the chemical composition of different dental amalgam restorations, providing information that can be used to determine the time period during which a deceased person has lived. The hypothesis is based on the premise that dental amalgam alloys consist of different elements and that the use of the various alloys was introduced and abandoned at certain times.

### Key measures for improvement

By incorporating this information along with the time of tooth eruption, it may be possible to narrow down the possible time frame during which the deceased person has lived.

## Materials and methods

### Brief description of context

The study was performed at the laboratory premises of the Nordic Institute of Dental Materials (NIOM) in May–June 2023. The use of amalgam was approved by the Norwegian Environment Agency (ref. 2022/4155) and dispensation from the use of mercury was given for research purposes. The dental amalgam was handled in accordance with the guidelines and regulations for laboratory settings. The three first-authors prepared the amalgam samples and performed the analyses under guidance of the last author. Qualified personnel assisted with the SEM–EDS analyses.

### Process of gathering information

Six previously commonly used amalgams were selected (Table 1); four conventional silver amalgams and two copper amalgams. Of the silver amalgams, three were defined by the producer to be high-copper amalgams and one as a low-copper amalgam. All amalgams were mixed according to the producer's instruction manuals. The amalgams were then transferred to 5 mm high cylindrical plastic moulds with a diameter of 3.5 mm and condensed using separate amalgam condensers (pluggers) for each brand of amalgam to avoid contamination between the different samples. A total of five samples were prepared of each amalgam brand.

**Table 1:** Results from the SEM–EDS-analysis (mean value (standard deviation)) of each amalgam test sample and information on amalgam alloy compositions as stated by the manufacturers. Deviations from stated compositions may be due to differences in the analytical substrate (powder versus set amalgam)

		Detected elements in wt% (with standard deviations)					
Brand	Manufacturer	Cu	Ag	Sn	Cd	Hg	Alloy type
Ultracap S	Dentsply Sirona	20.6 (1.05)	9.4 (0.43)	20.5 (1.52)	0.3 (0.15)	49.2 (2.06)	High-copper
*Info from manufacturer*		*18.4*	*16.8*	*17.4*	*0.0*	*47.4*	
Cavex AV Alloy	Cavex	7.9 (0.42)	31.9 (0.89)	12.4 (0.37)	0.4 (0.27)	47.4 (1.23)	High-copper
*Info from manufacturer*		*11.8*	*22.2*	*15.0*	*0.0*	*50.7*	
ANA Admix	Nordiska Dental	17.6 (1.87)	15.9 (1.10)	18.4 (1.07)	0.4 (0.15)	47.8 (3.97)	High-copper
*Info from manufacturer*		*	*	*	*	*	
Dispersalloy	Coltene	7.1 (0.50)	31.1 (1.00)	10.9 (0.30)	0.5 (0.28)	50.4 (1.39)	Low-copper
*Info from manufacturer*		*5.9*	*34.1*	*8.9*	*0.0*	*50.5*	
Cupromuc	Merz & Co	22.1 (2.80)	0.2 (0.63)	1.4 (0.79)	0.9 (0.40)	75.3 (1.85)	Copper–amalgam
*Info from manufacturer*		28.7	0.4	0.0	0.6	70.0	
AMDS**	Unknown**	22.1 (2.37)	0.01 (0.03)	0.8 (0.49)	0.0 (0.00)	77.0 (2.23)	Copper–amalgam
*Info from manufacturer*		*	*	*	*	*	

*The exact alloy composition of the amalgam used was not possible to obtain from the manufacturer

**AMDS: Aмaльгaмa мeднaя для cтoмaтoлoгии, (Amalgama mednaja dlja stomatologii); name of the manufacturer and alloy composition was not possible to identify

The amalgam samples were allowed to set overnight. The surface of each block was then polished in a polishing device (Struers Knuth Rotor, sandpaper #500 and #1200) under running water to obtain a smooth and unoxidized surface immediately prior to analysis.

**Fig. 1:**
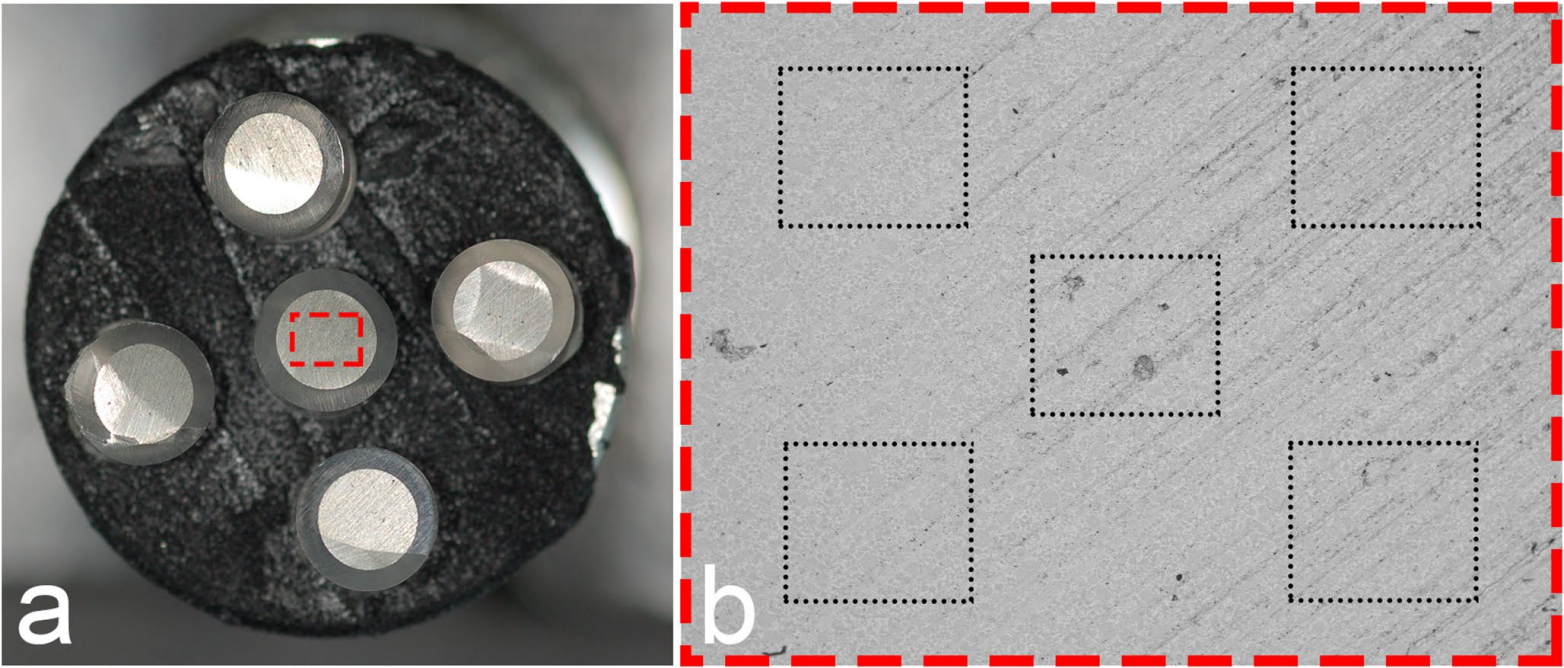
**a**) Photo of five samples prepared of each amalgam brand mounted on the view board prior to analysis. **b**) Photo of the position of the five predefined fields in each test sample used for the SEM/EDS readings

Each surface was analysed with a scanning electron microscope (Hitachi TM4000 Plus, Hitachi High-Technologies Corporation, Tokyo, Japan) at × 200 magnification and accelerating voltage of 15 kV at five different (not overlapping) sites as shown in Fig. 1, giving 25 readings for each amalgam brand. The position of the five fields of interest for SEM–EDS-readings was defined in the software prior to the analyses, and the same predefined squares were used consecutively for all samples in the project. Elements in the samples were quantified using the Bruker Quantax 75, Nano CmbH, Berlin, Germany, and the software Espirit Compact, Hitachi High Technologies Europa Gmb.

## Results

### Analysis and interpretation

The results of the SEM–EDS analysis of the six dental amalgams are presented in Table 1.

The SEM–EDS analyses reveal differences in composition among the six brands of amalgams. For comparison, Table 1 also includes a detailed overview of the contents and proportion of each element for each amalgam composition as provided by the manufacturers.

The SEM–EDS analyses identified noticeable differences in the contents and composition of the four conventional silver amalgams compared to the two copper amalgams. The two latter contained very low or non-detectable amounts of silver and tin. Surprisingly, small amounts of cadmium were detected in all amalgams even though only one amalgam (Cupromuc) listed cadmium as part of its elemental composition.

## Discussion

This study aimed to explore if scanning electron microscope (SEM) with SEM–EDS elemental analysis could be used to determine the chemical composition of different dental amalgam restorations. As the results show, the two copper amalgams were easily distinguishable from the conventional amalgams due to their lack of silver (Ag). This corresponds with the traditional and historical composition of copper amalgam, which typically consists of 60–70% mercury, 30–40% copper, and no silver [17]. In contrast, the conventional amalgams contained a large amount of silver, varying from 9–32%. Hence, while the discovery of human remains containing teeth with amalgam restorations presupposes that the person has lived after the introduction of dental amalgams in the 19^th^ century, the detection of a copper amalgam restoration implies that the individual lived during a period when this particular amalgam type was in use. This helps determining a timeframe when the person has lived. By combining this information with the time of tooth eruption, the possible time period the person has lived may be narrowed down to an even shorter period. For example, if a deceased person was discovered in Norway with a copper amalgam filling in a first molar, it implies that the tooth must have been filled before 1981 when the use of copper amalgam was phased out in Norway. Consequently, the person must have been at least six years old at that time, excluding all missing individuals born in the years after 1975.

Our study also showed the possibility not only to distinguish between conventional silver amalgams and copper amalgams, but also between the different conventional silver amalgams (low-copper vs. high-copper). However, the alignment observed between the SEM–EDS results and the composition according to the manufacturers, was not strong enough to differentiate the samples from different manufacturers. This could be because the information from the manufacturer were based on the composition of the alloy only whereas our analyses were after adding mercury, trituration, condensation and setting. In low-copper amalgams, both the gamma-1 phase, consisting of silver and mercury, and the gamma-2 phase, consisting of tin and mercury, are encapsulated within an unreacted gamma phase of both tin and silver following the curing process. In high-copper amalgams, the dominant phases post-curing are the gamma-1 phase and a copper-tin phase known as the eta-phase [2]. The composition of phases present on the surface area, where the SEM–EDS analysis is conducted, will influence the results.

Our results were based on laboratory-made specimens that had not been exposed to the oral cavity or post-mortem environmental effects. Following trituration, an oxide layer forms on the surface when the amalgam interacts with the surrounding environment. A study by Hanawa et al. from 1987 showed that a surface layer was formed within 20 min after trituration. This layer had a somewhat different composition than the remaining alloy [18]. To avoid such changes in surface mass concentrations of elements affecting the results, the surfaces of the specimens in this study were polished to remove the oxide layer immediately before the SEM–EDS analyses. This was based on the presumption that the underlying amalgam was mixed homogeneous to a certain degree. It is also advisable to polish amalgam restorations taken from human teeth before SEM–EDS-analysis in forensic cases, to avoid this possible bias.

Another important factor to consider is the impact of different environmental effects on amalgam fillings in human remains. For example, charred remains subjected to high temperatures are not uncommon in forensic cases. When dental amalgam is exposed to elevated temperatures, it undergoes several alterations in surface texture and structure, primarily influenced by the melting points of the constituent metals within the alloy [19]. Of particular interest in this study is the possible alteration of the silver content. Previous studies have observed the formation of gaseous bubbles within a temperature range of 200 °C to 400 °C that arise when mercury evaporates [20]. The evaporated mercury drives silver particles to the surface to form a superficial nodule, often referred to as “silver bullets”. As temperatures reach 800 °C to 1000 °C, the nodules disappear because of silver reaching a melting point and evaporates. Nevertheless, the risk of misinterpreting thermally altered conventional silver amalgams as copper amalgam is minimal. This is due to the distinctive macroscopic surface morphology characterized by spheres composed of either copper or tin, which form as the temperature decreases [19]. It is also unlikely that all silver evaporates. Thus, SEM–EDS analyses should be reliable even in charred human remains, given that the temperature has not been so high that the restoration melts completely [21].

In an anthropology-based study by Zelic et al. from 2013, dental restorations were chemically analysed to assess when the deceased persons could had lived. The chemical analyses were performed using inductively coupled plasma optical emission spectrometry (ICP-OES) on six dental filling materials, of which three were amalgams. Two of the amalgam fillings were identified as silver amalgam, while the third contained almost exclusively copper and mercury and no silver, and was suggested to be a copper amalgam [22]. The ICP-OES method is a procedure that requires the samples to be ionized in a high-temperature plasma. The resulting ions emit characteristic wavelengths of light, which are used to identify and quantify the elements of a sample. ICP-OES method analyses the material’s composition as bulk, since the measurement is performed on a homogenized sample dissolved in an appropriate acid. Such analyses therefore provide an average concentration measurement, whereas SEM–EDS analyses the surface area of the sample matching the electron beam’s size. Notably, SEM–EDS is a non-destructive analytical method, and sample preparation is swift and straightforward compared to the dissolution process required when performing ICP-OES. However, ICP-OES has a low detection limit, allowing it to detect elements at the ppm level. This makes it particularly effective for trace element analysis [23]. With a potential detection limit of 0,1–0,5 wt.% for most elements, SEM–EDS is more suitable to provide qualitative chemical analyses of elements [24]. However, in cases where such detection limits are acceptable, such as in the present study, SEM–EDS is a faster and cheaper method than ICP-OES. In addition, a study conducted by Alomary et al. in 2012 demonstrated a strong correlation between the data acquired through ICP-OES and those by SEM–EDS when assessing their ability to determine the quantitative composition of metals in sea sediments [25].

Previous studies have shown that copper amalgams may contain the toxic heavy metal cadmium [17]. This metal could theoretically serve as a parameter to distinguish copper amalgam from traditional silver amalgam. However, the SEM–EDS results presented in Table 1 surprisingly detected presence of cadmium in all test samples, which contradicts the compositions reported by the manufacturers. According to the ISO standard for dental amalgam alloy constituents above 0.1 wt% are to be declared by the manufacturers (ISO 24234:2021). An explanation for the inconsistencies might be misinterpretation of cadmium for another element as concentrations observed were close to the detection limit of SEM–EDS (0.1–0.5 wt%,). Also, depending on the sample matrix and the settings of the SEM–EDS instrument, there might be interferences or overlaps in X-ray emissions that lead to the false detection of cadmium. Incorrect calibration of the system could also lead to the misidentification of elements in the sample [26]. However, it cannot be ruled out that the amalgams may indeed contain cadmium unless the samples are analysed by method with lower detection limits like ICP-OES.

Using amalgam composition for our purpose is not exempt from limitations, the foremost being the obvious prerequisite to perform the proposed technique that teeth with amalgam restorations must be present. Also, SEM technology must be part of the medicolegal facilities. Nevertheless, as SEM is a common instrument in many laboratories at least in developed countries, the accessibility of this method makes it an easy applicable alternative when amalgam fillings are present. For instance, does the National Criminal Investigation Service in Norway possess a SEM. Furthermore, the instrument is more cost-efficient, easier to use, and less time-consuming than many other methods, such as radiocarbon testing and ICP-OES, not to mention that it is a non-destructive method. The authors do not know how feasible SEMs are for medicolegal use in countries outside Scandinavia (i.e. developing countries) but, if necessary, specimens of amalgam restorations could easily be sent to a laboratory for analysis.

Another limitation is the challenge of identifying the precise year of the copper amalgam phase-out in Norway. Copper amalgam was used for a long time, although its use was restricted in 1981 by the Norwegian Directorate of Health. A survey in 1994 revealed the ongoing utilization of the material by some dental practitioners, despite regulatory directives [11]. Nevertheless, it is reasonable to conclude that most Norwegian dentists adhered to recommendations from the Norwegian Directorate of Health and discontinued the use of copper amalgam in 1981. The present study has not been able to verify when other countries than Norway and Sweden abandoned the use of copper amalgam, if discontinued at all. While conventional silver amalgam has predominantly been phased out or subjected to restrictions in numerous countries, its use persists in certain areas. In some countries, this also includes copper amalgam. “Pyrax Polymars”, an Indian company, still supplies and exports copper amalgam. Although its usage may be limited, the Indian Dental Academy still considers it a usable alternative [9]. Thus, the technique presented in this study is mainly relevant for Scandinavian countries where regulations for the use of copper amalgam are known. Nevertheless, for all we know, this situation may be similar in numerous other countries, and it should not be too difficult or time-consuming to investigate when the use of copper amalgam was discontinued in the pertinent country if the technique is consider used.

### Strategy for change

This study demonstrated that SEM–EDS was useful to distinguish copper amalgam from conventional silver amalgam and that it might be possible to distinguish high-copper silver amalgam from low-copper ones. This information may be used to determine the time period during which a deceased person has lived, allowing the legal authorities to narrow down the search for reference material (ante mortem data) by excluding from the search missing persons with permanent teeth that erupted in the years after use of copper amalgam was discontinued.

### Next steps

Resin composites, which are frequently used by dentists today, have undergone numerous developments to enhance their properties. Over the years, there have been advancements such as reducing the size of filler materials, refining surface treatment, modifying monomer structure, and polymerization reactions, among other adjustments. Further research can be conducted using SEM–EDS to try to distinguish between different types and brands of composites.

## Declarations

### Structure of manuscript

The manuscript is structured according to the EQUATOR guidelines for quality improvement studies, section “A new structure for quality improvement reports” (Moss F, Thompson R. A new structure for quality improvement reports. Qual Health Care 1999;**8**:76| Qual Saf Health Care 2004;**13**:6–7).

### Data availability

Data sets generated during the current study are available from the corresponding author and/or the Nordic Institute of Dental Materials (NIOM) on reasonable request.
